# IL-33 promotes double negative T cell survival via the NF-κB pathway

**DOI:** 10.1038/s41419-023-05766-4

**Published:** 2023-04-05

**Authors:** Xiaojing Sun, Chunpan Zhang, Fanqi Sun, Shuxiang Li, Yaning Wang, Tingting Wang, Li Li

**Affiliations:** 1 Department of International Medical Center, Beijing Friendship Hospital, Capital Medical University, Beijing, China; 2 Department of Infectious Diseases, Beijing Friendship Hospital, Capital Medical University, Beijing, China; 3 Capital Medical University Forth Clinical School, Beijing, China; 4 Liver Research Center, Beijing Friendship Hospital, Capital Medical University, Beijing, China; 5 Beijing Key Laboratory of Translational Medical On Liver Cirrhosis, Beijing, China; 6 National Clinical Research Center for Digestive Diseases, Beijing, China

**Keywords:** Immune cell death

## Abstract

IL-33, which is a crucial modulator of adaptive immune responses far beyond type 2 response, can enhance the function of several T cell subsets and maintain the immune homeostasis. However, the contribution of IL-33 to double negative T (DNT) cell remains unappreciated. Here, we demonstrated that the IL-33 receptor ST2 was expressed on DNT cells, and that IL-33 stimulation increased DNT cells proliferation and survival in vivo and in vitro. Transcriptome sequencing analysis also demonstrated that IL-33 enhanced the biological function of DNT cells, especially effects on proliferation and survival. IL-33 promoted DNT cells survival by regulating Bcl-2, Bcl-xl and Survivin expression. IL-33-TRAF4/6-NF-κB axis activation promoted the transmission of essential division and survival signals in DNT cells. However, IL-33 failed to enhance the expression of immunoregulatory molecules in DNT cells. DNT cells therapy combined with IL-33 inhibited T cells survival and further ameliorated ConA-induced liver injury, which mainly depended on the proliferative effect of IL-33 on DNT cells in vivo. Finally, we stimulated human DNT cells with IL-33, and similar results were observed. In conclusion, we revealed a cell intrinsic role of IL-33 in the regulation of DNT cells, thereby identifying a previously unappreciated pathway supporting the expansion of DNT cells in the immune environment.

## Introduction

Double negative T (DNT) cells are peripheral mature T cells that express CD3 but not CD4, CD8 or natural killer (NK) T cell markers [1]. Although these cells represent a small subpopulation of approximately 1–5% of T lymphocytes in peripheral blood, they have been suggested to possess immunoregulatory functions in an antigen-specific manner [2]. Young NOD mice which exert a high proportion of DNT cells in spleen are potentially resistant to diabetes [3]. DNT cells can suppress the T and B cells proliferation and attenuate Graft-versus-host disease [4]. In addition, some studies have identified that DNT cells play an important role in immune surveillance against pathogens, including *Francisella tularensis* [5], *Leishmania major* [6], and influenza virus [7]. Moreover, our previous studies demonstrated that DNT cells converted from CD4^+^ T cells could also suppress excessive inflammatory responses and help to maintain immune homeostasis in liver injury [8, 9], type 1 diabetes [10] and allergic asthma [11].

IL-33, a cytokine that belongs to the IL-1 superfamily, was first identified in human tissues in 2003. Primarily, it activates many immune cell subsets involved in innate immunity and type 2 immune responses. Meanwhile, studies in recent several years have uncovered important roles of IL-33 in the activation of immune cells such as Th1 cells, NK cells, CD8^+^ T cells, neutrophils, macrophage, infection and chronic inflammation, as well as the immunoregulatory function of regulatory T (Treg) cells [12]. However, the effects of IL-33 on DNT cells are still unknown.

In this study, we demonstrated that IL-33 could promote the proliferation and inhibit the apoptosis in mouse and human DNT cells through the TRAF4/6-NF-κB signaling, which was accompanied by increased Bcl-2, Bcl-xl and Survivin expression. While, DNT cells combined with IL-33 could exert a greater effect on maintaining immune system homeostasis due to the proliferative effect of IL-33.

## Materials and methods

### Animals

Male C57BL/6 (*H-2*^*b*^), DBA/2 (*H-2*^*d*^), and B6D2F1 (*H-2*^*b/d*^) mice were purchased from Beijing Vital River Laboratory (Beijing, China). Male CD45.1 congenic C57BL/6 mice were obtained from the Jackson Laboratory. The mice were kept under specific pathogen-free conditions at the animal facilities of Beijing Friendship Hospital, and all animal protocols were approved by the Institutional Animal Care and Ethics Committee. The IACUC number was 21-2010.

### Reagents and antibodies

Mouse and human recombinant IL-33 (580506, 581804) were obtained from Biolegend (CA, USA). ConA was obtained from Sigma-Aldrich (C2010, St. Louis, MO, USA). GreenNuc^TM^ Caspase-3 Assay Kit for live cells was obtained from Beyotime (C1168M, Shanghai, China). Mouse T cell activation/expansion kit was purchased from Miltenyi Biotec (130-093-627, Auburn, CA). The BrdU staining kit (8817-6600-42) and Cell Trace^TM^ Violet (CTV) Cell Proliferation Kit (C34557) for flow cytometry were obtained from Thermo Fisher Scientific (MA, USA). Fluorochrome-conjugated antibodies to mouse/human CD3, CD4, CD8, NK1.1/CD56, CD25, CD11b, TCRγδ, B220, TER119, Ki67, ST2, CD45.1, CD45.2, H-2D^d^, Bcl-2 for flow cytometry were obtained from Biolegend. Antibodies to mouse Bcl-xl, Survivin and phospho-IKKα/β were obtained from Cell Signaling Technology (MA, USA). Antibodies to mouse NF-κB and IκBα were purchased from Abcam (MA, USA). All the details of reagents and antibodies were listed in Table S1.

### Conversion of DNT cells in vitro

Converted DNT cells from CD4^+^ T cells were obtained as previously described [13]. Briefly, purified CD4^+^CD25^−^ T cells derived from spleens and lymph nodes of C57BL/6 mice were incubated with DBA/2 mature dendritic cells (mDCs) and recombinant mouse IL-2 (50 ng/ml) (200-02, PeproTech, NJ, USA). Seven days later, DNT cells were sorted with a FACS Aria II cell sorter (BD Biosciences, CA, USA).

### Restimulation of DNT cells in vitro

DNT cells (2 × 10^5^/well) were restimulated with anti-mouse CD3/CD28 beads with or without IL-33 (10/20/50/100/200/300/500 ng/ml) in a 96-well round-bottom culture plates for 24 h or 48 h. The cells were harvested for staining, real-time PCR and RNA sequencing.

### Restimulation of DNT cells in vivo

B6D2F1 (*H-2*^*b/d*^) recipient mice (*n* = 10) received CD45.1 positive DNT cells (3 × 10^6^) (purity >97%) by intravenous route. Then, the mice were randomly divided into two groups. One group of mice (*n* = 5) were received BrdU (100 μg/day) and recombinant IL-33 (1.5 μg/100 μl/day) via intraperitoneal injection, the other group of mice (*n* = 5) were received BrdU (100 μg/day) and PBS (100 μl/day) for 3 days. The B6D2F1 mice were killed and CD45.1 positive DNT cells proliferation in the spleen were measured by BrdU incorporation and Ki67 staining according to the manufacturer’s instructions.

### In vitro suppression assays

DNT cells (2 × 10^5^/well) were restimulated with or without IL-33 (20 ng/ml, 50 ng/ml and 100 ng/ml) in the presence of anti-mouse CD3/CD28 beads for 48 h, according to previous study [14]. CTV-labeled naive CD45.1^+^CD4^+^CD25^−^ T cells (2 × 10^5^/well) isolated from C57BL/6 congenic mice were incubated with mDCs (0.5 × 10^5^/well) in 96-well plates. Differentially treated DNT cells (0.5 × 10^5^/well) were added to the mixed lymphocyte reaction (MLR). After five days, the apoptosis and proliferation were detected with Annexin V and CTV staining, respectively.

### Transcriptome sequencing analysis

DNT cells were stimulated with or without IL-33 (50 ng/ml) for 48 h, and total RNA was extracted. Library preparation was constructed with NEBNext^®^ Ultra^TM^ RNA Library Prep Kit for Illumina^®^ (NEB, MA, USA) according to the manufacturer’s instructions. High-throughput sequencing was performed using the Illumina NovaSeq 6000 in Annoroad Gene Technology. Differential expressed genes (DEGs) analysis between two groups was performed with *p*adj value less than 0.05 and |log2(fold change)| ≥0.585. Then, Gene Ontology (GO) and Kyoto Encyclopedia of Genes and Genomes (KEGG) pathway enrichment analysis of DEGs were performed in the DAVID database (https://david.ncifcrf.gov/tools.jsp). Gene set enrichment analysis (GSEA) was performed using the OmicStudio tools (https://www.omicstudio.cn/tool). RNA sequencing data have been deposited for public access in the National Center for Biotechnology Information (NCBI) Gene Expression Omnibus (GEO) database under the GEO accession number GSE206839.

### Human DNT cells expansion in vitro

TCRα/β^+^CD4^−^CD8^−^ double negative T cells were isolated from human peripheral blood mononuclear cells (PBMCs) by a double negative T cell isolation kit (130-092-614, Miltenyi Biotec). Then, 2 × 10^5^ cells/well were cultured with purified anti-human CD3 antibody (2 µg/ml) (317302, Biolegend) in X-VIVO^TM^ 15 Serum-free cell medium (04-418Q, Lonza, Oregon, USA) supplemented with human recombinant IL-2 (50 ng/ml) (212-12, PeproTech). Fourteen days later, DNT cells were harvested and restimulated with or without recombinant human IL-33 (20/50/100 ng/ml) in a 96-well plate-bottom culture plates for 24 h or 48 h. The cells were harvested for staining and RNA extraction. This study protocol was approved by the Human Institutional Review Board of Beijing Friendship Hospital (2022-P2-058-01).

### Concanavalin A-induced liver injury model

The animal model of Concanavalin A (ConA)-induced liver injury was performed as previously described [9]. Briefly, CD45.1 congenic C57BL/6 mice were randomly divided into five groups (*n* = 5 in control group; *n* = 5 in ConA group; *n* = 5 in ConA+IL-33 group; *n* = 5 in ConA+DNT group; *n* = 5 in ConA+DNT + IL-33 group). To study the protective function of DNT cells and IL-33, mice were treated with CD45.2 positive converted DNT cells (3 × 10^6^/mouse, i.v. injection) and/or IL-33 (1.5 μg/mouse, i.p. injection) following ConA treatment (15 mg/kg body weight, i.v. injection). The mice of control group were treated with equal-volume PBS. After ConA administration for 24 h, the mice were sacrificed. Serum alanine aminotransferase (ALT) activity was determined with an alanine aminotransferase assay kit (C009-2, NJJC Bio Inc, Nanjing, China) according to the manufacturer’s instructions. Liver samples were fixed with 4% paraformaldehyde and then stained with hematoxylin-eosin (H&E) staining. The degree of histopathological liver damage was evaluated with the Inshak scoring system [15] and the evaluator was blinded to treatment.

### Preparation of hepatic mononuclear cells for Flow cytometric analysis

Under deep anesthesia, livers were perfused with 30 ml of normal saline through the left cardiac apex, as described in previous study [16]. Next, the liver was split into small pieces and digested with 0.01% type IV collagenase (C5138-1G, Sigma) at 37 °C. After 30 min, the liver mixture was dissociated with a gentleMACS^TM^ dissociator, followed by filtration through a 70 µm nylon cell strainer. To purify hepatic mononuclear cells (MNCs), the mixture was suspended in 30% Percoll (Cytiva, MA, USA) and centrifuged at 500×*g* for 5 min. The cell pellet was used to detect the different subsets of intrahepatic MNCs by flow cytometry.

### Flow cytometric analysis

Flow cytometry was performed with a FACS Aria II flow cytometer. FlowJo v10.4 software was used to analyze the data.

### RNA extraction and real-time PCR

Total RNA was obtained with TRI reagent (T9424-200ML, Sigma). To obtain complementary DNA, reverse transcription was performed by using the PrimeScript RT Reagent Kit (RR036A, Takara, Kyoto, Japan). For quantitative PCR, 7500 Fast Real-time System (Applied Biosystems) was used with SYBR Green Master Mix (11202ES08, Yeasen, Shanghai, China). The specific primers were detailed in Table S2.

### Statistical analysis

Graphical presentations and statistical analyses were performed with Prism 8 (Graph Pad). No sample was excluded from the analyses. Data were first analyzed using Shapiro-Wilk test to know if they were normally distributed. Data of two groups following a normal distribution were analyzed using an *F*-test to know the equality of variances between groups, and analyzed using two-tailed unpaired Student’s *t* test with or without Welch’s correction. Data of two groups for non-normal distribution were analyzed using Mann–Whitney test. One-way ANOVA or repeated measures One-way ANOVA with post hoc Dunnett’s, Turkey or Holm-Sidak’s test, depending on the recommendation by prism for normal distribution were performed for multiple comparisons. Values were expressed as the mean ± standard deviation (SD). *P* values <0.05 were considered significant. All experiments were performed at least two or three times. The sample size was estimated from our preliminary experiments or from previous reports [9, 17].

## Results

### IL-33 promoted proliferation and inhibited apoptosis of DNT cells

**Fig. 1:**
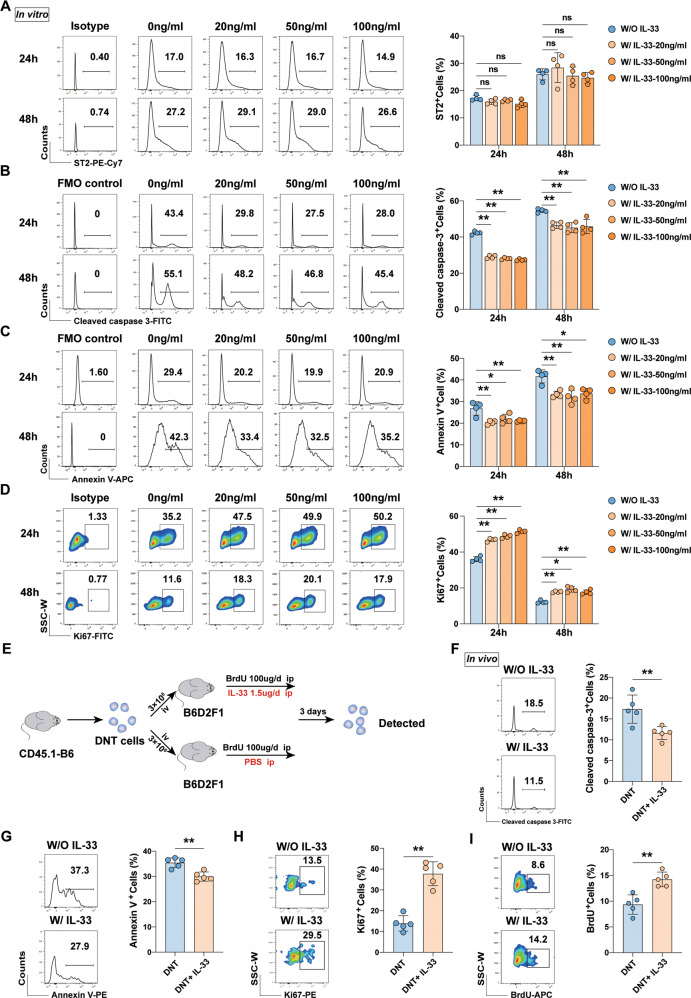
IL-33 regulated the survival of DNT cells. Converted DNT cells were stimulated with or without IL-33 for 24 h and 48 h in vitro, and representative flow cytometry plots and statistical analysis of ST2 (**A**), cleaved caspase-3 (**B**), Annexin V (**C**), and Ki67 expression (**D**) levels were performed (*n* = 4/group). **E** Flowchart showing the adoptive transfer mode. B6D2F1 mice were received a total of 3 × 10^6^ CD45.1 positive DNT cells by tail vein injection. At the same time, IL-33 was administrated daily by intraperitoneal injection for 3 days. Representative flow cytometry plots and statistical analysis of cleaved caspase-3 (**F**), Annexin V (**G**), Ki67 (**H**), and BrdU (**I**) expression in CD45.1 positive DNT cells (*n* = 5/group). Data in **A**–**D** were analyzed using repeated measures One-way ANOVA post hoc Turkey’s test. Data in **F**–**I** were analyzed using unpaired Student’s *t* test without Welch’s correction (equal variances). Data are represented as the mean ± SD. *P < 0.05, **P < 0.01, ns no significance.

Purified CD4^+^CD25^−^ T cells were incubated with mDC and recombinant mouse IL-2. Seven days later, mixed cells were obtained and incubated with fluorescent-labeled anti-mouse antibodies against CD3, NK1.1, CD4 and CD8. Converted DNT cells (CD3^+^NK1.1^−^CD4^−^CD8^−^) were sorted with FACS Aria II cell sorter (Fig. S1). To investigate the effects of IL-33 on DNT cells, we cultured DNT cells under anti-mouse CD3/CD28 beads stimulation in vitro in the presence of different concentrations of IL-33, and examined the expression of the IL-33 receptor ST2 on the cell surface. Flow cytometry analysis showed that ST2 was expressed on DNT cells (Figs. 1A and S2A), and the apoptosis and proliferation were revealed by Annexin V and Ki67 staining at 24 h and 48 h, respectively. After optimal exogenous IL-33 stimulation (20, 50, and 100 ng/ml), cleaved caspase-3 expression and apoptosis of DNT cells were decreased (Figs. 1B, C and S2B, C), and the proliferation rate was significantly increased (Figs. 1D and S2D), although the expression of ST2 did not change significantly (Fig. 1A).

Additionally, to assess the role of IL-33 in DNT cells proliferation and apoptosis in vivo, B6D2F1 (*H-2*^*b/d*^) recipient mice received 3 × 10^6^ converted CD45.1 positive DNT cells by tail vein injection (Fig. 1E) along with daily administration of BrdU (100 μg/mouse) and IL-33 (1.5 μg/mouse) for 3 days. CD45.1 positive DNT cells were detected (gating strategy was shown in Fig. S3A). Consistent with the in vitro results, the apoptosis rate of CD45.1 positive DNT cells was decreased (Fig. 1F, G), while the proliferation rate of CD45.1 positive DNT cells was increased in the IL-33 administration group (Figs. 1H, I and S3B). These in vitro and in vivo data confirmed a vital role of IL-33 during the survival of DNT cells.

### IL-33 promoted DNT cell survival by regulating Bcl-2, Bcl-xl, and Survivin expression

**Fig. 2:**
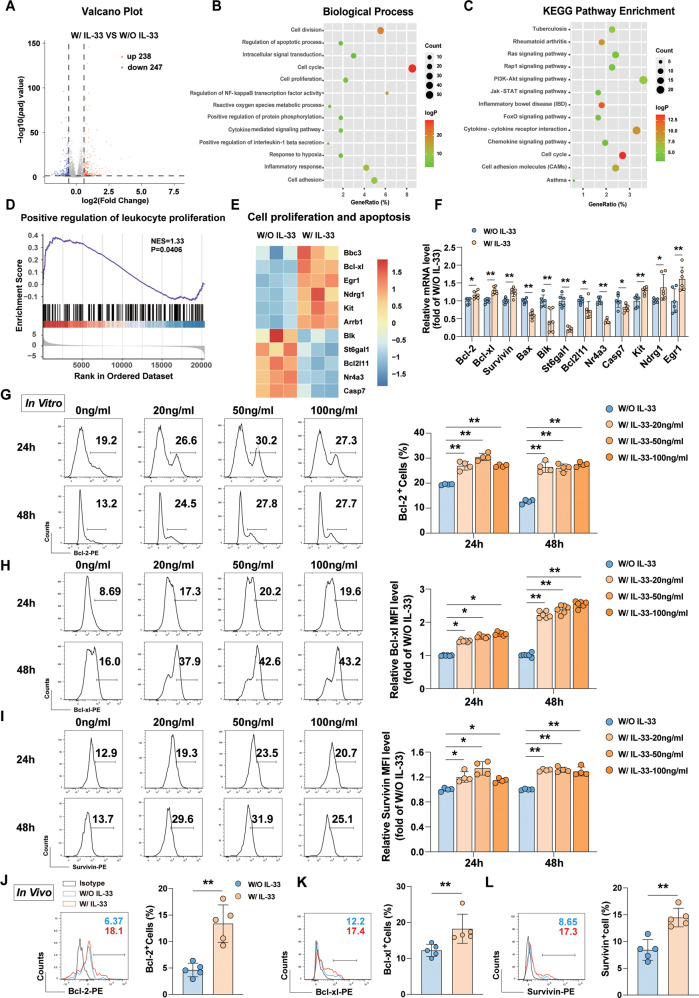
IL-33 promoted DNT cells survival by regulating Bcl-2, Bcl-xl, and Survivin expression. **A**–**E** Bulk mRNA sequencing data of DNT cells that were treated with or without IL-33 (50 ng/ml for 48 h). **A** A volcano plot in which each dot represents a gene with |log2(fold change)| ≥ 0.585 and *p*adj value < 0.05 (upregulated (238) and downregulated (247)). Enriched GO (**B**) and KEGG (**C**) pathway analyses were performed based on DEGs. **D** GSEA plot showing highly significant enrichment of positive regulation of leukocyte proliferation in DNT cells with IL-33 stimulation. **E** A heatmap of genes related to cell proliferation and survival. **F** Real-time PCR results showing the mRNA expression of the significantly changed genes related to cell survival after IL-33 stimulation of DNT cells (*n* = 6/group). DNT cells were stimulated with or without IL-33 for 24 h and 48 h in vitro. Representative flow cytometry plots and statistical analysis of Bcl-2 (**G**), Bcl-xl (**H**), and Survivin (**I**) levels (*n* = 4 or 6/group). Representative histogram plots and quantification of Bcl-2 (**J**), Bcl-xl (**K**), and Survivin (**L**) levels in adoptively transferred DNT cells in B6D2F1 recipient mice (*n* = 5/group). Data in **G**, **I** were analyzed using repeated measures One-way ANOVA post hoc Turkey’s test. Data in **F**, **J** and **L** were analyzed using unpaired Student’s *t* test without Welch’s correction (equal variances). Data in **K** were analyzed using Mann–Whitney test. Data are represented as the mean ± SD. *p < 0.05, **p < 0.01. See also Fig. S4.

To further characterize the role of IL-33 in DNT cell function, we performed bulk RNA sequencing of DNT cells with or without IL-33 stimulation (50 ng/ml for 48 h). A total of 485 DEGs were finally obtained with |log2(fold change)| ≥0.585 and *p*adj value <0.05, and a volcano map of DEGs was shown in Fig. 2A. GO (biological processes) and KEGG pathway enrichment analysis of the DEGs showed that the differences were mainly involved in the cell division, the regulation of apoptosis and proliferation, inflammatory response and cytokine mediated signaling pathway, among others (Fig. 2B, C), which suggested that IL-33 had extensive effects on DNT cell survival and biological functions. In addition, various signaling pathways, such as Janus kinase-signal transducer and activator of transcription (JAK-STAT) and nuclear factor kappa-B (NF-κB) pathways (Fig. 2B, C), were involved. GSEA showed that the positive regulation of leukocyte proliferation was increased (NES = 1.33, *P* = 0.0406; Fig. 2D). Moreover, compared with untreated DNT cells, DNT cells stimulated with IL-33 revealed downregulated proapoptotic genes (*Bik*, *Bcl2l11*, *St6gal1*, *Nr4a3*, *and Casp7*), and upregulated antiapoptotic genes (*Bcl-xl*, *Kit*, *Ndrg1*, *and Egr1*) (Fig. 2E). We further confirmed these changes in the related genes by real-time PCR and flow cytometry. As shown in Figs. 2F–I and S4, IL-33 enhanced Bcl-2, Bcl-xl, and Survivin expression in DNT cells, while significantly downregulated expression *St6gal1, Nr4a3, Bax*, *and Bik* (Fig. 2F), which indicated that these molecules participated in IL-33-mediated regulation of DNT cells survival. Furthermore, we examined the expression of antiapoptotic proteins in CD45.1 positive DNT cells from B6D2F1 recipient mice. Bcl-2 (Fig. 2J), Bcl-xl (Fig. 2K), and Survivin (Fig. 2L) were upregulated after IL-33 administration. These experiments revealed that IL-33 regulated DNT cell survival through antiapoptotic proteins.

### IL-33 promoted DNT cell survival via the TRAF4/6-associated NF-κB signaling pathway

**Fig. 3:**
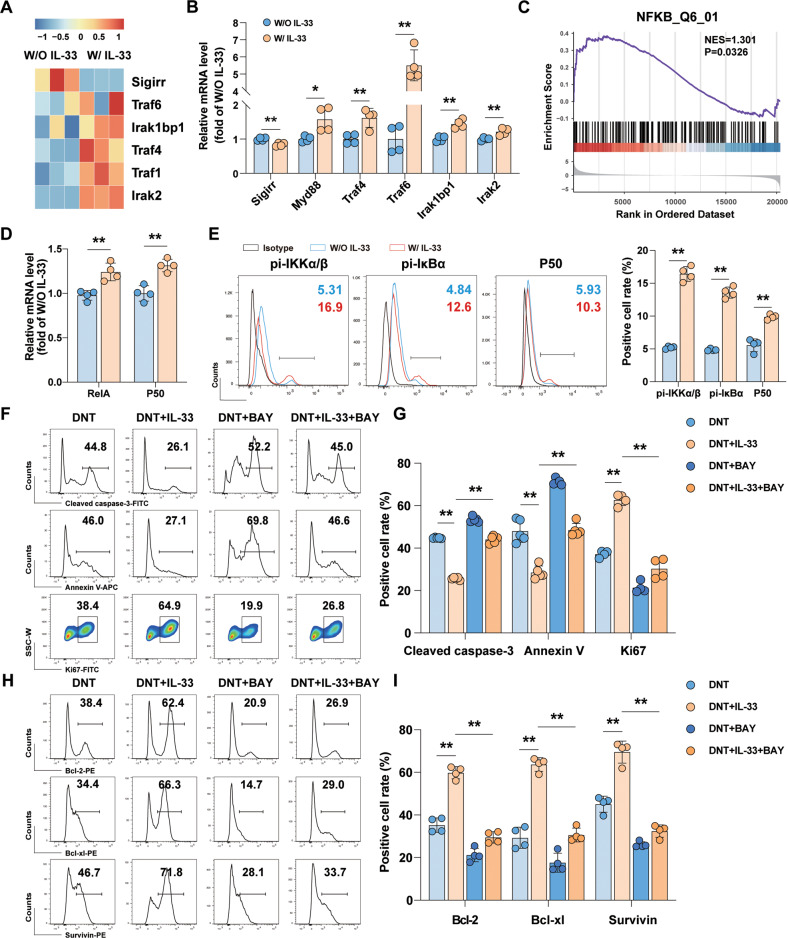
IL-33 promoted DNT cells survival via the NF-κB signaling pathway. **A** The data were presented as a heatmap exhibiting differential gene expression related to IL-33 signaling. **B** Genes mRNA expression was confirmed by real-time PCR after IL-33 stimulation (*n* = 4/group). **C** GSEA of a NFKB_Q6_01 gene set in DNT cells stimulated with IL-33 compared with control DNT cells. **D** Relative mRNA expression of NF-κB signaling pathway-related genes (*n* = 4/group). **E** Flow cytometric and statistical analysis of P50 protein expression, and the phosphorylation of IKKα/β and IκBα protein expression in DNT cells with or without IL-33 stimulation for 48 h (*n* = 4/group). Representative flow cytometry plots (**F**) and statistical analysis (**G**) of cleaved caspase-3^+^, Annexin V^+^ and Ki67^+^ DNT cells percentages after incubation with an NF-κB signaling inhibitor (BAY 11-7082) with or without IL-33 stimulation for 48 h (*n* = 4 or 5/group). Representative flow cytometry plots (**H**) and statistical analysis (**I**) of the Bcl-2^+^, Bcl-xl^+^, and Survivin^+^ DNT cells percentages after incubation with an NF-κB signaling inhibitor (BAY 11-7082) with or without IL-33 for 48 h (*n* = 4/group). Data in **B**, **D** and **E** were analyzed using unpaired Student’s *t* test with or without Welch’s correction depending on the *F*-test results. Data in **G** and **I** were analyzed using repeated measures One-way ANOVA post hoc Turkey’s test. The data are represented as the mean ± SD. *p < 0.05, **p < 0.01. See also Fig. S5.

IL-33 interacts with its receptor complex, leading to the recruitment of the adapter MyD88 (myeloid differentiation factor 88), IRAK (IL-1 receptor associated kinase), and TRAF6 (tumor necrosis factor receptor associated factor 6). As shown in Fig. 3A, B, transcriptome sequencing and mRNA analysis showed that the expression of single immunoglobulin interleukin 1 related receptor (*Sigirr*), which is an IL-33 activities inhibitor, was downregulated after IL-33 stimulation. Intracellular regulators such as *Myd88, Irak2, Irak1bp1, Traf4, and Traf6* were upregulated. GSEA showed that NFKB_Q6_01 was related to the regulation of IL-33 on DNT cells (NES = 1.301, *P* = 0.0326, Fig. 3C). We also demonstrated changes in *RelA* and *P50*, which are key members in the canonical NF-κB signaling pathway, by real-time PCR (Fig. 3D). Moreover, flow cytometry analysis displayed that IL-33 stimulation increased IKKα/β and IκBα phosphorylation levels as well as P50 expression (Fig. 3E).

To confirm the role of NF-κB signaling in IL-33-mediated regulation of DNT cells proliferation and survival, the cultures were treated with or without an NF-κB signaling inhibitor (BAY 11-7082, 0.5 μM) and IL-33 (50 ng/ml) to form the DNT, DNT+BAY, DNT+IL-33, and DNT+BAY+IL-33 groups. As an aforementioned result, cleaved caspase-3 expression and the apoptosis percentage of DNT cells were decreased, and an increase in the proliferation percentage was induced by IL-33 stimulation. BAY 11-7082 application displayed an impaired impact from antiapoptotic effect to antiapoptotic molecules expression. Cleaved caspase-3 expression and Annexin V positive rates were increased (Figs. 3F, G and S5A, B) as well as a decrease of Bcl-2, Bcl-xl and Survivin (Figs. 3H, I and S5C, D) in the presence of an NF-κB signaling inhibitor. These data thus demonstrated the functional relevance of the TRAF4/6-associated NF-κB signaling pathway in IL-33-mediated regulation of DNT cell proliferation and survival, which was accompanied by an increase of Bcl-2, Bcl-xl and Survivin expression in DNT cells.

### IL-33 stimulation did not enhance the expression of immunoregulatory molecules in DNT cells

**Fig. 4:**
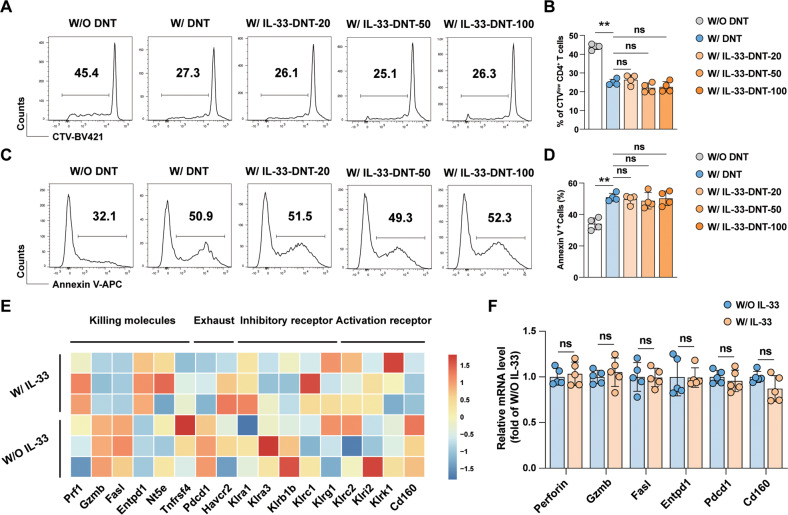
IL-33 did not enhance the expression of immunoregulatory molecules in DNT cells. DNT cells (2 × 10^5^/well) were pre-stimulated with or without IL-33 (20/50/100 ng/ml) for 48 h. Then, the culture medium was discarded, and DNT cells (0.5 × 10^5^/well) pre-stimulated with or without IL-33 were incubated with naive CD45.1^+^CD4^+^ T cells (2 × 10^5^/well) from C57BL/6 congenic mice and mDCs (0.5 × 10^5^/well) for 5 days, respectively. Representative flow cytometric (**A**) and statistical results (**B**) showing the proliferation of CD4^+^ T cells examined, as determined by CTV on the 5th day (*n* = 4/group). Representative histogram plots (**C**) and quantification (**D**) of the apoptosis rates of CD4^+^ T cells, as examined via Annexin V staining (*n* = 4/group). Heatmap (**E**) and quantitative real-time PCR-based confirmation (**F**) of the expression of leukocyte-mediated cytotoxicity related genes in IL-33-stimulated DNT cells (*n* = 5/group). Data in **B** and **D** were analyzed using repeated measures One-way ANOVA post hoc Tukey’s test. Data in **F** were analyzed using unpaired Student’s *t* test without Welch’s correction (equal variances). Data are represented as the mean ± SD. **p < 0.01, ns no significance.

To further clarify the impact of IL-33 on DNT cells function, we examined the immunosuppressive effect of IL-33-stimulated DNT cells on CD4^+^CD25^−^ T cells in vitro. In contrast to the simple MLR system, the proliferation of CD4^+^CD25^−^ T cells in DNT cell group was decreased. However, compared with that in DNT cell group, proliferation of CD4^+^CD25^−^ T cells did not change significantly in different doses of IL-33 pre-stimulated DNT cell groups (Fig. 4A, B). We then examined the apoptosis rate of CD4^+^ T cells, and pre-stimulation of DNT cells with IL-33 did not cause an increase in the frequency of Annexin V^+^ cells among CD4^+^ T cells (Fig. 4C, D). Furthermore, we compared the levels of cytotoxicity related genes between the two groups. *Perforin*, *Granzyme B*, and *Fasl*, which are the main effector molecules of DNT cells [13], were not altered in IL-33-pre-stimulated DNT cells. The expression of genes encoding potential inhibitory receptors, such as *Klra1*, *Klra3*, *Klrb1b*, *Klrc1*, and *Klrg1* [18], and activation receptors, such as *Klrc2*, *Klri2*, *Klrk1*, and *Cd160* [19] (Fig. 4E, F), in two groups were similar. These data revealed that IL-33 stimulation has little effect on immunoregulatory molecule expression in DNT cells.

### IL-33 enhanced DNT cell function by promoting its expansion

**Fig. 5:**
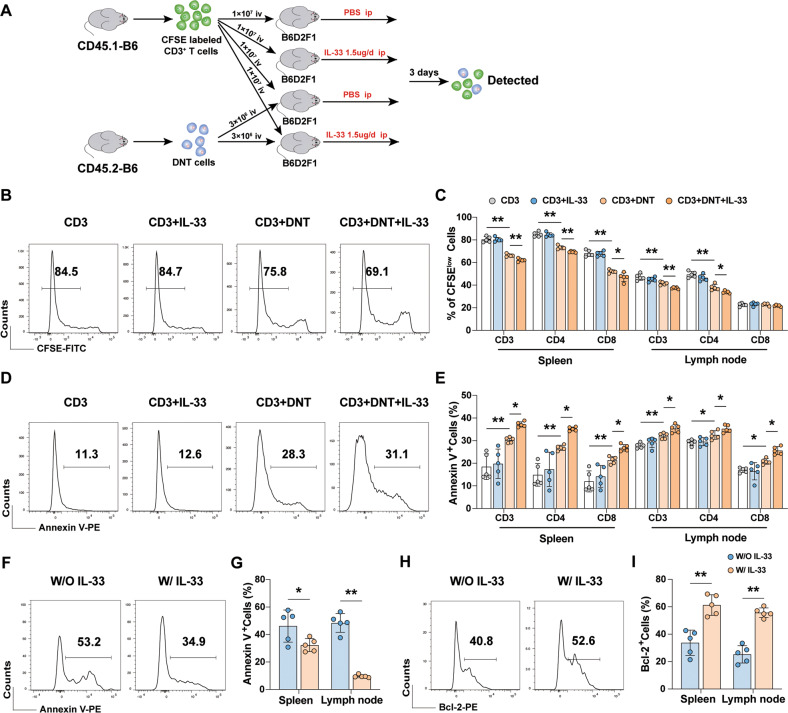
IL-33 enhanced DNT cell function by promoting its expansion. **A** Flowchart showing suppression test method in vivo. **B** Representative flow cytometric results showing the proliferation of adoptively transferred CD4^+^ T cells, as examined by CFSE in the spleen. **C** Statistical analysis of the percentage of proliferating CD3^+^ T cells, CD4^+^ T cells and CD8^+^ T cells in spleen and lymph nodes in each group. Representative flow cytometric results of apoptosis in adoptively transferred CD4^+^ T (**D**) and DNT cells (**F**) in the spleen. **E**, **G** Statistical analysis of the apoptosis rates in the spleen and lymph nodes in each group. **H** Representative flow cytometric results of Bcl-2 expression in adoptively transferred DNT cells in the spleen. **I** Quantification of Bcl-2 expression levels in spleen and lymph nodes in each group. Data in **C** and **E** were analyzed using One-way ANOVA post hoc Holm-Sidak’s test. Data in **G** and **I** were analyzed using unpaired Student’s *t* test with or without Welch’s correction. The data are represented as the mean ± SD, *n* = 5/group. *p < 0.05, **p < 0.01.

Because of the immunosuppressive effects of IL-33-pre-stimulated DNT cells on CD4^+^CD25^−^ T cells in vitro were not changed, IL-33 and DNT cells were added to the MLR system together. It is interesting that the proliferation of CD4^+^CD25^−^ T cells was further decreased and the apoptosis rate was increased in response to DNT cells with IL-33 co-stimulation compared with DNT cells only group (Fig. S6). IL-33 may affect the proliferation and apoptosis in DNT cells (Fig. 1) but not CD4^+^CD25^−^ T cells (Fig. S6). To identify whether exogenous IL-33 co-stimulation could promote the cytotoxicity function of DNT cells in vivo, CFSE labeled CD45.1^+^CD3^+^ T cells and CD45.2^+^ DNT cells were adoptively transferred into B6D2F1 mice (Fig. 5A). After 3 days of IL-33 administration, adoptively transferred CD45.1^+^ T cells in the spleen and lymph nodes were examined (gating strategy was shown in Fig. S7A). As shown in Fig. 5B, C, the proliferation of CD45.1^+^CD3^+^ T cells, CD45.1^+^CD4^+^ T cells and CD45.1^+^CD8^+^ T cells were inhibited by DNT cells. Furthermore, DNT cells combined with IL-33 induced a lower proliferation rate than the other groups. The apoptosis rates of CD45.1^+^ T cells were consistent with the results of CFSE analysis, as determined by flow cytometry (Fig. 5D, E). Splenocytes or cells in lymph nodes were obtained and incubated with fluorescent-labeled anti-mouse antibodies against CD45.2, H-2D^d^, CD3, and NK1.1 (gating strategy was shown in Fig. S7B), and the apoptosis rates of adoptively transferred DNT cells (CD45.2^+^H-2D^d−^CD3^+^NK1.1^−^) were decreased (Fig. 5F, G). Bcl-2 expression levels were increased after IL-33 administration (Fig. 5H, I). These results suggested that IL-33 could enhance DNT cell function by promoting its survival in vitro *and* in vivo.

### DNT cell therapy combined with IL-33 could further ameliorate ConA-induced liver injury

**Fig. 6:**
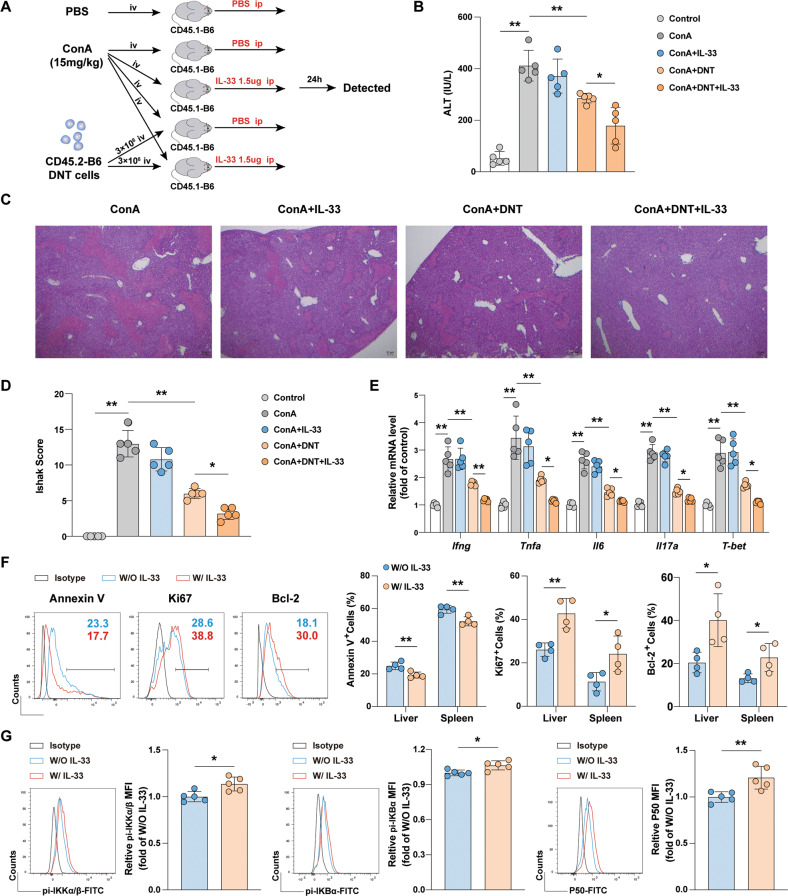
DNT cells therapy combined with IL-33 could further ameliorate ConA-induced liver injury. **A** Flowchart showing ConA, DNT cells and IL-33 administration in each group. **B** Serum ALT was measured in each group. **C** Representative H&E staining of the liver. **D** Liver pathological scores in each group. **E** Relative mRNA levels of *Ifng, Tnfa, Il6, Il17a*, and *T-bet* in liver tissues. **F** Representative histogram plots showing the quantification of Annexin V, Ki67 and Bcl-2 in CD45.2^+^ DNT cells in the liver and spleen in ConA+DNT and ConA+DNT+IL-33 groups. **G** Representative histogram plots and quantification of IKKα/β and IκBα phosphorylation and P50 protein expression in CD45.2^+^ DNT cells in the two groups. Data in **B** and **D** were analyzed using One-way ANOVA post hoc Tukey’s test. Data in **E** were analyzed using One-way ANOVA post Holm-Sidak’s test. Data in **F** and **G** were analyzed using unpaired Student’s *t* test without Welch’s correction (equal variances). The data are represented as the mean ± SD, *n* = 4 or 5/group. *p < 0.05, **p < 0.01.

Our previous study showed that DNT cells could ameliorate ConA-induced liver injury [9]. To further examine whether IL-33 could promote DNT cell-mediated maintenance of immune homeostasis in immune-mediated liver damage, we adoptively transferred converted CD45.2^+^ DNT cells to CD45.1 congenic C57BL/6 mice following with ConA and IL-33 administration (Fig. 6A). Consistent with previous observations, DNT cells significantly limited ConA-induced liver injury [9]. DNT cells combined with IL-33 further ameliorated liver injury compared with that in the DNT cells group. First, serum ALT levels were significantly decreased (Fig. 6B). Hepatocyte necrosis was also significantly reduced in the DNT cells combined with IL-33 group (Fig. 6C, D). The mRNA levels of the proinflammatory cytokines *Ifng*, *Tnfa*, *Il6*, and *Il17a* were also downregulated after DNT cells and IL-33 treatment in the liver tissue (Fig. 6E). The apoptosis and proliferation rates of adoptively transferred DNT cells in the liver and spleen were analyzed by Annexin V and Ki67 staining. With IL-33 administration, the apoptosis rates of DNT cells were decreased, and the proliferation rates and Bcl-2 expression were significantly increased (Fig. 6F). Flow cytometry analysis showed that IL-33 administration also increased the phosphorylation of IKKα/β and IκBα and the protein expression of P50 (Fig. 6G). We concluded that DNT cells could exert better effects on maintaining immune system homeostasis due to the proliferative effect of IL-33.

### IL-33 stimulation promoted proliferation and inhibited apoptosis in human DNT cells

**Fig. 7:**
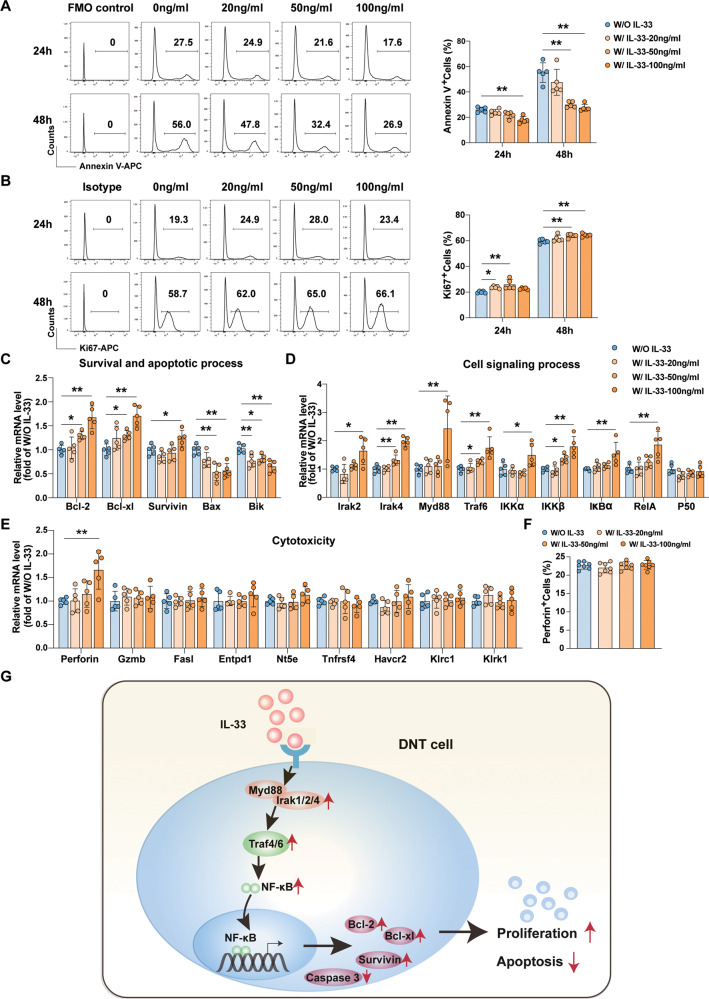
IL-33 stimulation promoted human DNT cell proliferation and survival in vitro. Human DNT cells were stimulated with or without IL-33 for 24 h and 48 h in vitro. Representative histogram plots and quantification of Annexin V (**A**) and Ki67 (**B**) levels in each group. Cell survival (**C**), cell signaling process (**D**), and cytotoxicity (**E**) related genes mRNA expression in response to IL-33 stimulation for 48 h. **F** Quantification of perforin expression levels by flow cytometric analysis in response to IL-33 stimulation for 48 h. **G** Proposed mechanism of IL-33 regulating DNT cells proliferation and survival. Data in **A**, **B** and **F** were analyzed using repeated measures One-way ANOVA post hoc Dunnett’s test. Data in **C**–**E** were analyzed using One-way ANOVA post hoc Dunnett’s test. The data are represented as the mean ± SD, *n* = 5/group. *p < 0.05, **p < 0.01.

To determine whether our findings were relevant to humans, DNT cells were isolated from PBMCs. After expansion in vitro, DNT cells were detected by flow cytometry (gating strategy was shown in Fig. S8). As shown in Fig. 7A, B, IL-33 stimulation significantly reduced the apoptosis rates and increased the proliferation rates of DNT cells at 24 h and 48 h. The expression of survival and apoptosis related genes, the TRAF-associated NF-κB signaling pathway and cytotoxicity related genes changes were similar to previous observations in mouse DNT cells. IL-33 promoted DNT cells survival by regulating the expression of antiapoptotic-related genes such as *Bcl-2*, *Bcl-xl*, and *Survivin* (Fig. 7C). IL-33-TRAF-NF-κB pathway mediated survival signals transmission in DNT cells (Fig. 7D). No significant difference was found in immunoregulatory molecules expression between DNT cells and IL-33 stimulated DNT cells (Fig. 7E, F).

## Discussion

IL-33 is a pleiotropic cytokine that enhances differentiation and expansion of Th1, Th2, CD8^+^ T cells, and Treg cells subsets with receptor complex [20–22]. Of note, the effect of IL-33 on regulatory cells expansion contributes to the maintenance of immune homeostasis [23, 24]. In this study, we presented evidence that IL-33 activities extended to DNT cells, a kind of T cell that mediated immune regulatory function.

Transcriptome sequencing analysis showed that IL-33 enhanced different biological functions of DNT cells, especially proliferation and survival. DNT cells exposed to IL-33 had higher proliferation rates and diminished apoptosis rates both in vitro and in vivo. Previous studies reported that IL-33 attenuated mast cells apoptosis primarily through Bcl-xl [25]. Moreover, IL-33 greatly enhanced CD8^+^ T cell survival by upregulating Bcl-2 expression [26]. Mechanistically, we found that IL-33 drove the expression of the antiapoptotic protein Bcl-2, Bcl-xl, and Survivin in DNT cells. Our previous study also demonstrated that Bcl-2, Bcl-xl, Bcl-2L11, and the cell cycle progression related protein Survivin participated in DNT cells survival regulation [27]. Therefore, the finding in this study is not only overlaps with the pathway by which IL-33 supports the survival of other cell types [28, 29], but is also consistent with our previous findings.

Membrane ST2 and IL-1R accessory protein (IL-1RAcP), which is a coreceptor shared with other IL-1 family members, are the main components of the IL-33 receptor complex [30]. The expression of ST2 was examined on Th2 cells, together with Th1 and cytotoxic T cells as well as Treg cells. We first demonstrated that ST2 was expressed on DNT cells. Other receptors may also play a key role in the conduction of IL-1 family cell signaling. With the results from real-time PCR examination, we found that *Sigirr* was downregulated, though the expression level of ST2 was stable. Sigirr serves a negative regulatory function on ILR and TLR-mediated pathways possibly by trapping IRAK1 and TRAF6, while the extracellular domain may interfere with heterodimerization of ST2 and IL-1RAcP [31]. Therefore, we hypothesized that the change in Sigirr expression could contribute to the signaling conduction in DNT cells. The Toll/IL-1 receptor (TIR) domains of IL-33 receptor binding to adapter molecules initiated the cell signaling transduction. We also detected the expression of classical intracellular adapter molecules at the mRNA level, and MyD88 and IRAK were upregulated by IL-33 stimulation. TRAF4 (tumor necrosis factor receptor associated factor 4), which is a member of the TRAF family, can modulate the function of Treg cells [32]. We found that not only TRAF4 but also TRAF6 was significantly overexpressed by IL-33 stimulation in DNT cells.

IL-33 and its receptors could alter the expression of genes which are correlated with activation of MAPKs and NF-κB cell signaling [33]. In our study, we confirmed the drastically increased levels of IKKα/β and IκBα phosphorylation, and p50 nuclear import in IL-33-stimulated DNT cells, accompanied with changes in Bcl-2, Bcl-xl and Survivin. Taken together, these findings demonstrated that it was most likely that IL-33 stimulation enhanced DNT cells survival with increasing expression of Bcl-2, Bcl-xl and Survivin due to TRAF4/6- NF-κB cell signaling.

IL-1 family members can not only promote effector T cell expansion [34] but also play an important role in the function of these cells [35]. Lucas et al. showed that IL-33 could activate Treg cells to suppress innate γδ T cell responses [36]. Recent study verified IL-33 promoted Treg cells function in intestinal tissues not only via enhancing transforming growth factor-β1 (TGF-β1) mediated differentiation but also providing a necessary signal for Treg cells accumulation and maintenance [24]. As a new kind of immunoregulatory cell, DNT cells highly express cytotoxic lymphocyte-related cytokines such as perforin and granzyme B. Studies have shown that DNT cells possess an immune regulatory function, capable of controlling anti-donor T cell responses in allo- and xenotransplantation through Fas-Fas ligand interaction [37]. Sequencing analysis and quantitative PCR results verified perforin, granzyme B and Fasl, as well as potential activation and inhibitory receptors expression of DNT cells were not elevated by IL-33 stimulation, and the immunosuppressive effect of DNT cells, pre-stimulated with IL-33, was not enhanced. However, DNT cells could further exaggerate cell death in proliferating CD4^+^ T cells when IL-33 was added together in vitro and in vivo. IL-33 is elevated in liver injury [38–41], and the IL-33/ST2 axis can prevent ConA-induced liver injury [42, 43]. Moreover, DNT cells therapy combined with IL-33 further ameliorated ConA-induced liver injury. Furthermore, the apoptosis rate of DNT cells was decreased, and Bcl-2 expression was increased by IL-33 administration. Therefore, we hypothesized that IL-33 could enhance DNT cells function by promoting expansion without directly influencing key immunoregulatory molecules. In addition, IL-33 elevation is found in allergic asthma [44], and DNT cells ameliorated allergic asthma [11] may partially by IL-33-induced expansion. However, the effect of IL-33 on ST2^+^ DNT cells is not clear and requires further in depth exploration.

In conclusion, our findings provided a new understanding of IL-33 and DNT cells that IL-33-mediated DNT cells proliferation and survival occurred via TRAF-NF-κB signaling, and was accompanied with increased Bcl-2, Bcl-xl, and Survivin expression (Fig. 7G). These findings may have important implications in DNT cells-based cell therapy in the future.

## Supplementary information

Supplementary figure

Supplementary table

Reproducibility checklist

## Data Availability

The analyzed data sets generated during the study are available from the corresponding author on reasonable request.
