# Supplementary material for: IL-33 promotes double negative T cell survival via the NF-κB pathway
Source: Cell Death Dis. 2023 Apr 5;14(4):242. doi: 10.1038/s41419-023-05766-4 (PMC10076344; doi:10.1038/s41419-023-05766-4)
Supplement: Supplementary file 1 — Supplementary figure [file 41419_2023_5766_MOESM1_ESM.docx]

**Supplementary Figures**

**
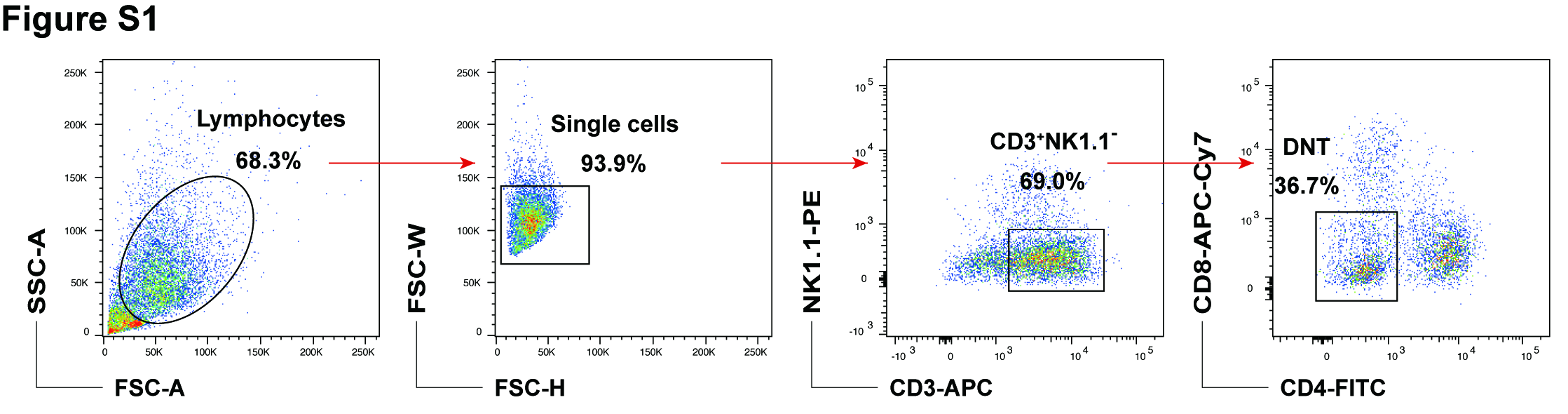
**

**Figure S1. Gating strategy of converted DNT cells.** Upon stimulation with mDCs and IL-2 in a mixed lymphocyte reaction, seven days later, CD4^+^CD25^−^ T cells showed a conversion to DNT cells. Converted DNT cells (CD3^+^NK1.1^−^CD4^−^CD8^−^) were sorted.


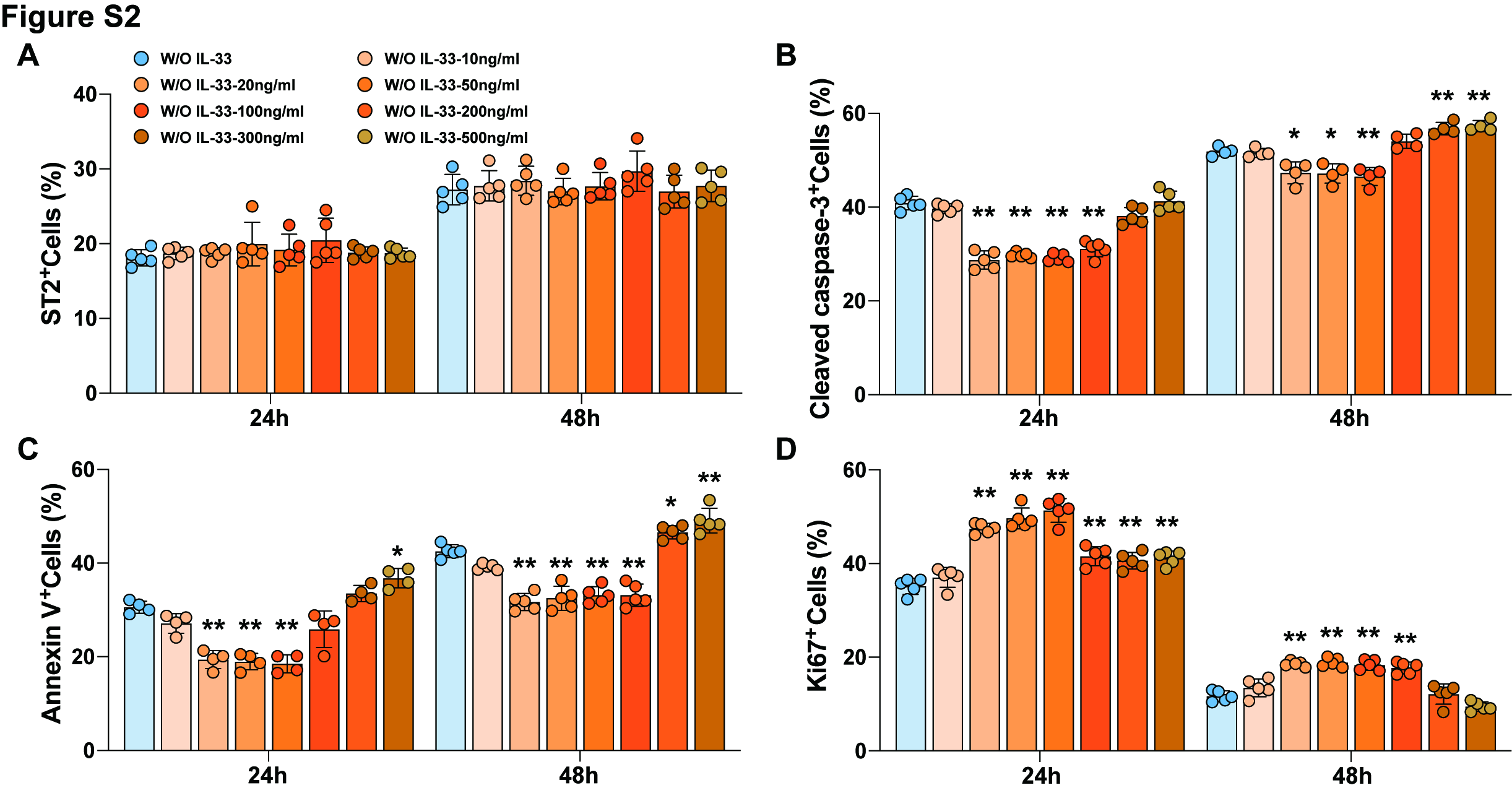


**Figure S2. IL-33 regulated the survival of DNT cells in different concentrations.** Converted DNT cells were stimulated with or without IL-33 for 24 h and 48 h *in vitro*. Statistical analysis of ST2 **(A)**, cleaved caspase 3 **(B)**, Annexin V **(C)** and Ki67 expression **(D)** levels were performed (n=4 or 5/group). Data in (**A-D**) were analyzed using repeated measures One-way ANOVA post hoc Turkey’s test. Data are represented as the mean ± SD. *P < 0.05, **P < 0.01.


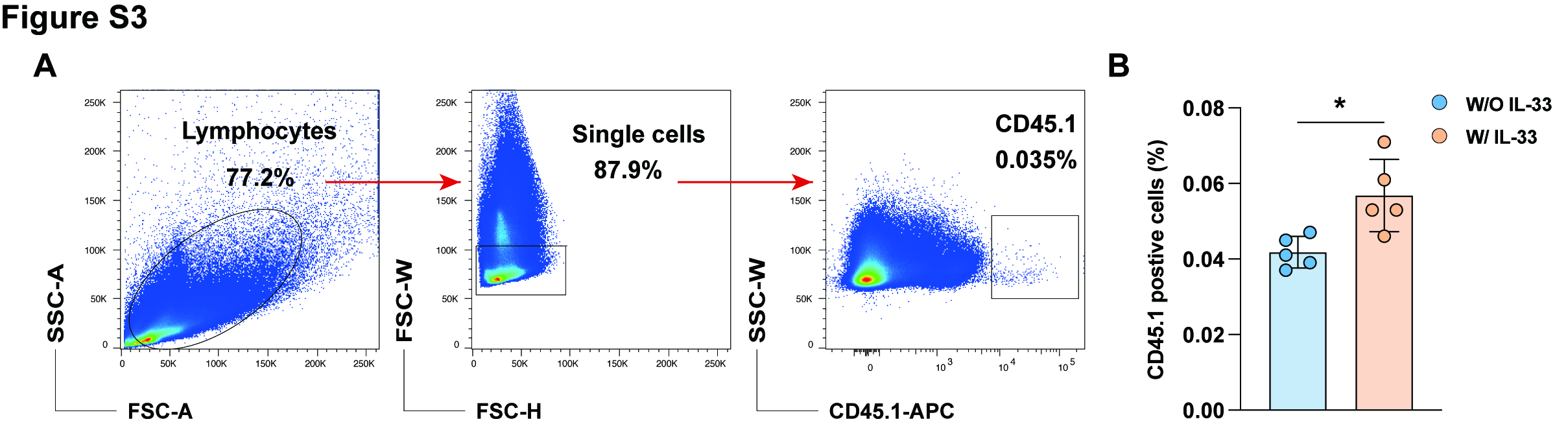


**Figure S3. Gating strategy and the percentage of adoptively transferred DNT cells between W/O IL-33 and W/ IL-33 groups. (A)** Splenocytes of B6D2F1 mice were stained with anti-mouse CD45.1 antibody, DNT cells (CD45.1^+^ cells) were gated. **(B)** Statistical analysis of the percentage of adoptively transferred DNT cells between W/O IL-33 and W/ IL-33 groups. Data in (**B**) were analyzed using unpaired Student’s *t* test without Welch’s correction (equal variances). Data are represented as the mean ± SD. *P < 0.05.


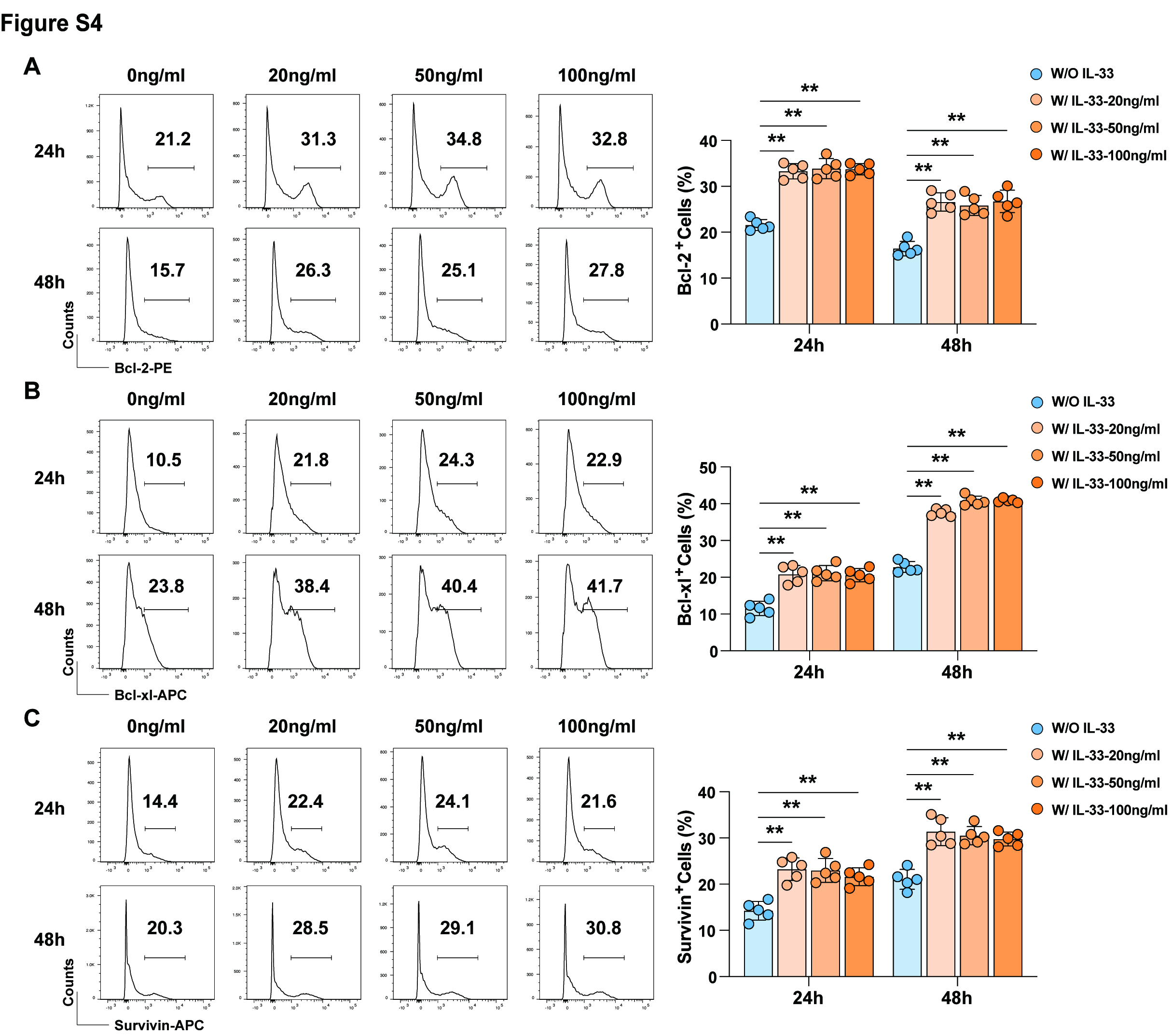


**Figure S4. IL-33 promoted Bcl-2, Bcl-xl and Survivin expression on DNT cells**. DNT cells were stimulated with or without IL-33 for 24 h and 48 h *in vitro*. Representative flow cytometry plots and statistical analysis of Bcl-2 (**A**), Bcl-xl (**B**), and Survivin (**C**) levels (n=5/group). Data in (**A-C**) were analyzed using repeated measures One-way ANOVA post hoc Turkey’s test. Data are represented as the mean ± SD. **P < 0.01.


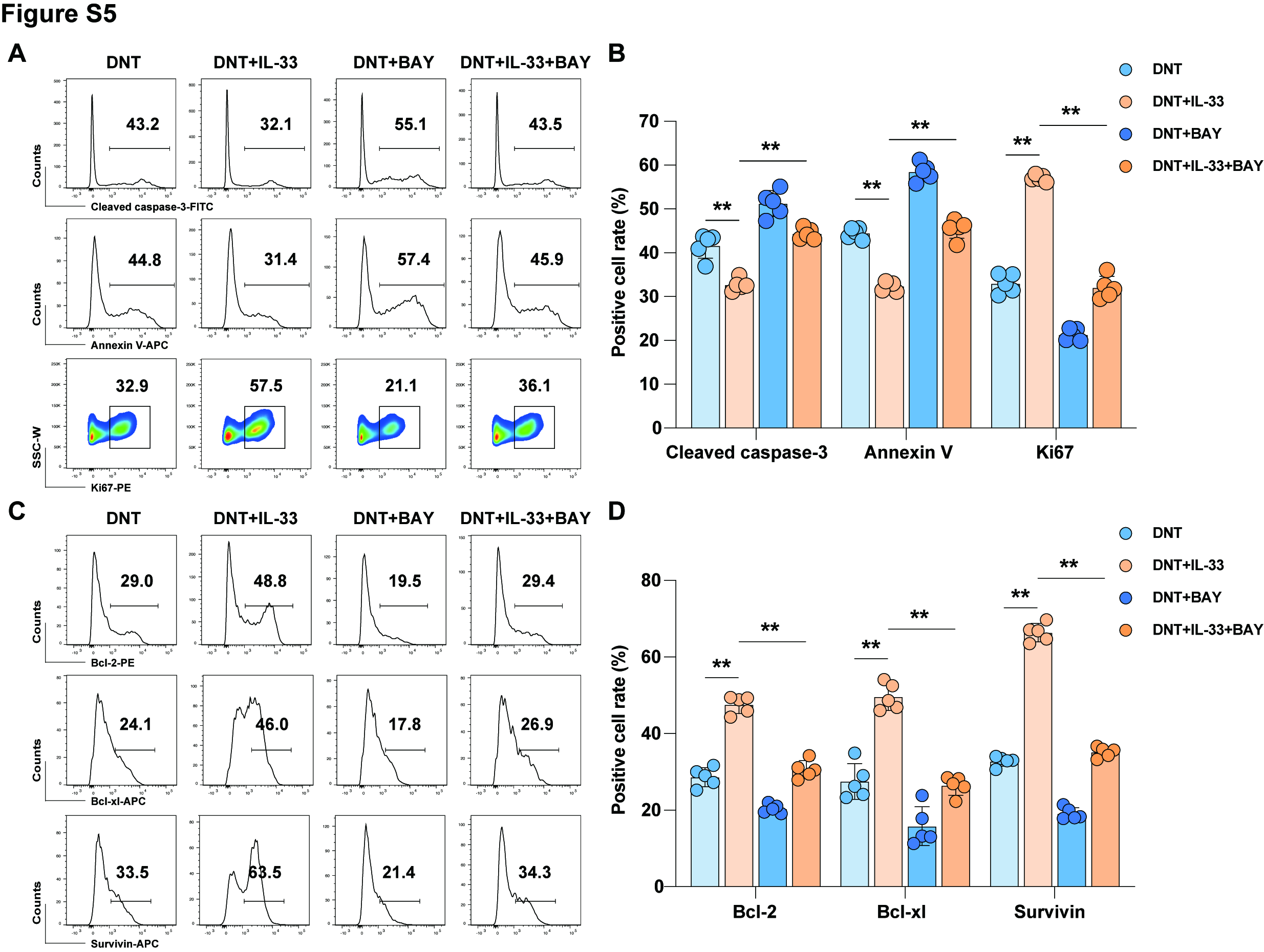


**Figure S5. IL-33 promoted DNT cells survival via** **the NF-κB signaling pathway**. Representative flow cytometry plots **(A)** and statistical analysis **(B)** of cleaved caspase-3^+^, Annexin V^+^ and Ki67^+^ DNT cells percentages after incubation with an NF-κB signaling inhibitor (BAY 11-7082) with or without IL-33 stimulation for 48 h (n=5/group). Representative flow cytometry plots **(C)** and statistical analysis **(D)** of the Bcl-2^+^, Bcl-xl^+^ and Survivin^+^ DNT cells percentages after incubation with an NF-κB signaling inhibitor (BAY 11-7082) with or without IL-33 for 48 h (n=5/group). Data in (**B and D**) were analyzed using repeated measures One-way ANOVA post hoc Turkey’s test. Data are represented as the mean ± SD. **P < 0.01.


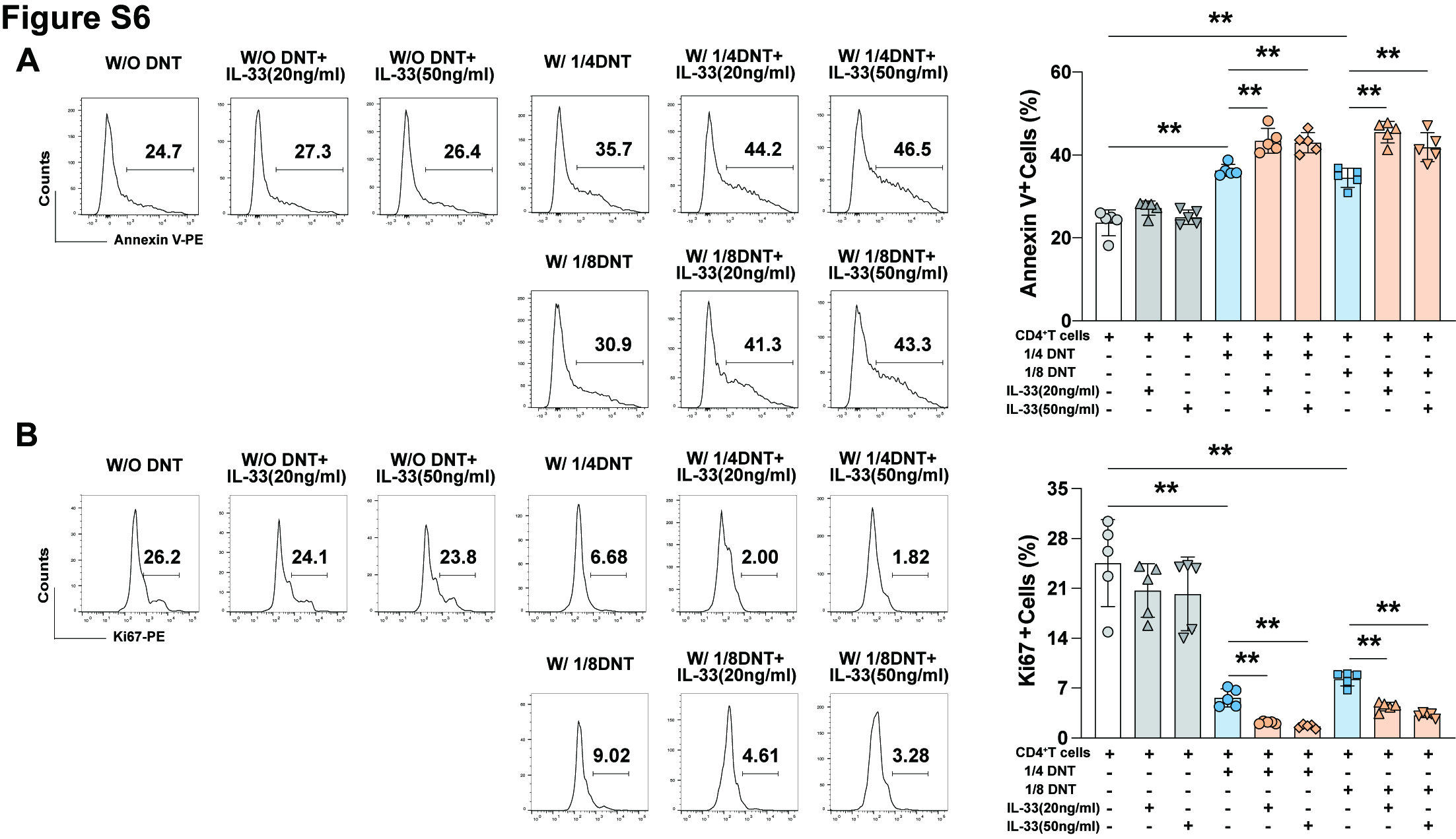


**Figure S6. Continuous IL-33 stimulation improved DNT cells suppression functions *in vitro*.** DNT cells and IL-33 were added to the MLR *in vitro*. **(A)** Representative histogram plots and quantification of CD4^+^CD25^−^ T cell apoptosis (Annexin V^+^) in each group on the 5th day. **(B)** Representative histogram plots and quantification of CD4^+^CD25^−^ T cell proliferation (Ki67^+^). Data in (**A** and **B**) were analyzed using repeated measures One-way ANOVA post hoc Holm-Sidak’s test. Data are represented as the mean ± SD, n=5/group. **p < 0.01.

**
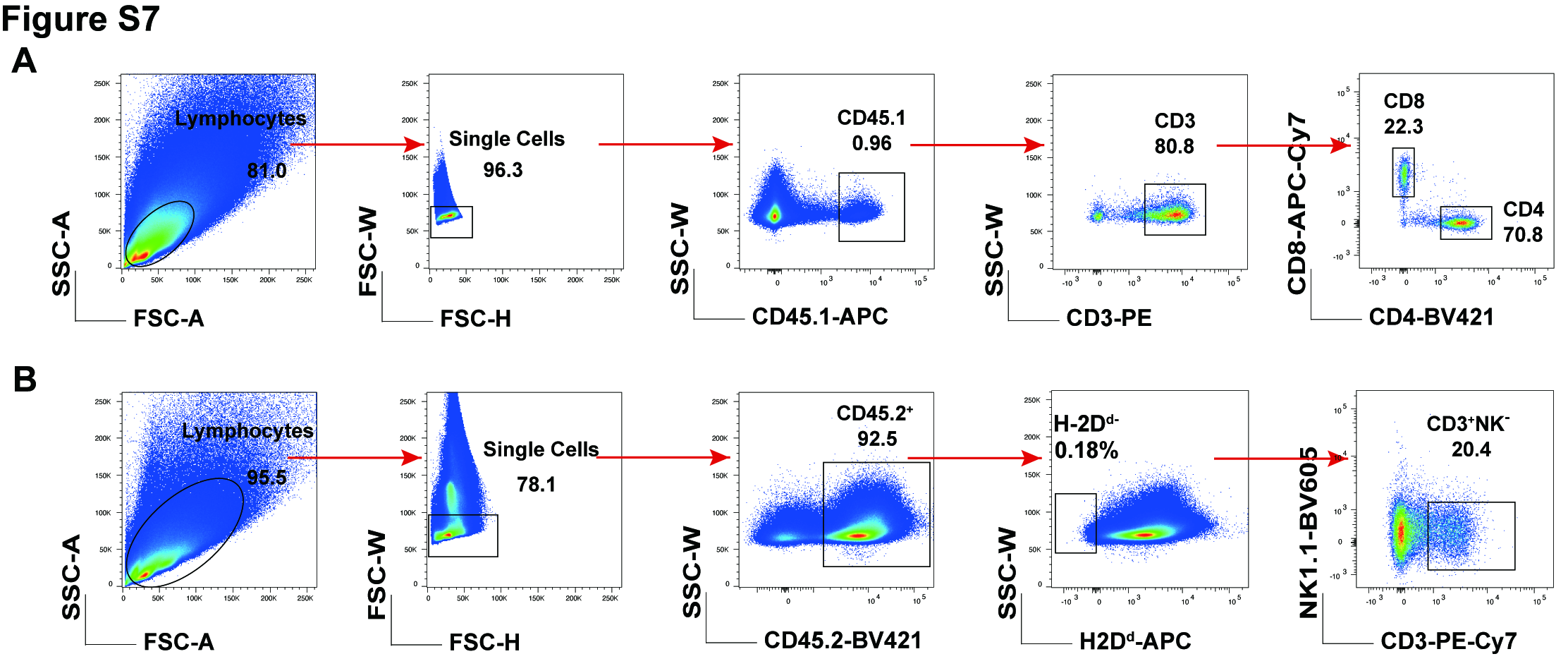
**

**Figure S7. Gating strategy of target cells in the spleen. (A)** Gating strategy of CD45.1^+^CD3^+^ T cells, CD45.1^+^CD4^+^ T cells and CD45.1^+^CD8^+^ T cells in the spleen. **(B)** Gating strategy of adoptively transferred DNT cells in the spleen.


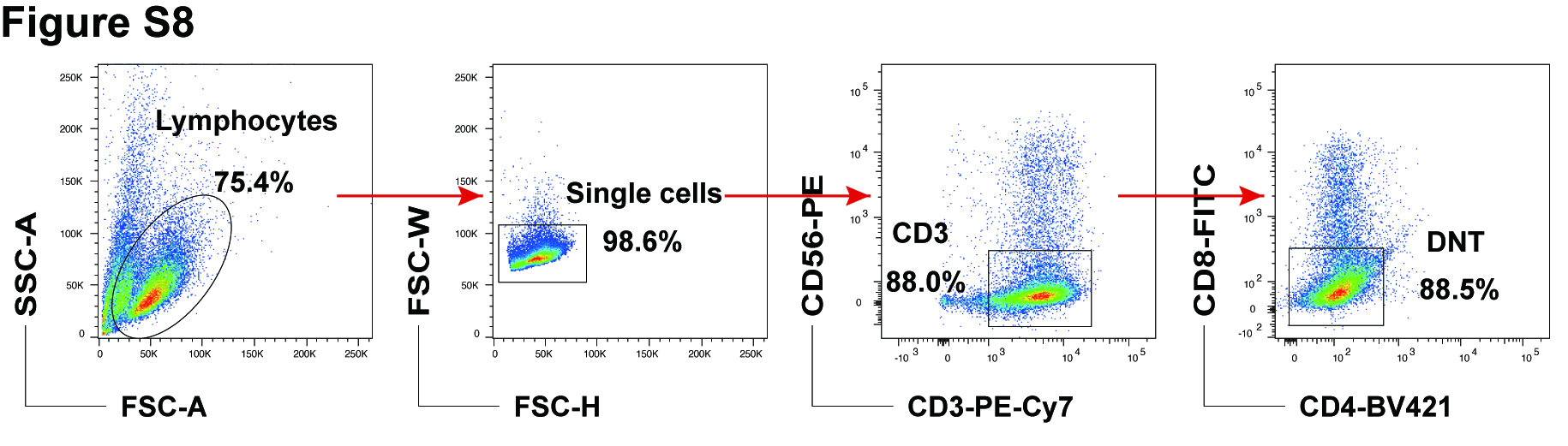


**Figure S8. Gating strategy of human DNT cells.** DNT cells were isolated from PBMCs by a double negative T cell isolation kit. After expansion *in vitro*, DNT cells were gated and tested.
