# Supplementary material for: IL-33 promotes double negative T cell survival via the NF-κB pathway
Source: Cell Death Dis. 2023 Apr 5;14(4):242. doi: 10.1038/s41419-023-05766-4 (PMC10076344; doi:10.1038/s41419-023-05766-4)
Supplement: Supplementary file 2 — Supplementary table [file 41419_2023_5766_MOESM2_ESM.docx]

**Supplemental Tables**

***Table S1 Reagents and antibodies used in this study***

| Source | Reagents and antibodies | Identifier |
| --- | --- | --- |
| Biolegend | Anti-mouse CD3 (PE/Cyanine7) | 100220 |
| (San Diego, CA, USA) | Anti-mouse CD3 (PE) | 100206 |
|  | Anti-mouse CD4 (Precp-Cyanine5.5) | 100432 |
|  | Anti-mouse CD4 (BV421) | 100438 |
|  | Anti-mouse CD8a (PE) | 100708 |
|  | Anti-mouse CD8a (APC/Cyanine7) | 100714 |
|  | Anti-mouse NK1.1 (PE) | 156504 |
|  | Anti-mouse NK1.1 (BV605) | 108740 |
|  | Anti-mouse CD25 (PE) | 102008 |
|  | Anti-mouse CD11b (PE) | 101208 |
|  | Anti-mouse TCRγδ (PE) | 107508 |
|  | Anti-mouse B220 (PE) | 103208 |
|  | Anti-mouse TER119 (PE) | 116208 |
|  | Annexin V (PE) | 640947 |
|  | Annexin V (APC) | 640941 |
|  | Anti-mouse Ki67 (FITC) | 652410 |
|  | Anti-mouse Ki67 (PE) | 652404 |
|  | Anti-mouse ST2 (PE/Cyanine7) | 145315 |
|  | Anti-mouse Bcl-2 (PE) | 633508 |
|  | Anti-mouse CD45.1 (APC) | 110714 |
|  | Anti-mouse CD45.1 (PE/Cyanine7) | 110730 |
|  | Anti-mouse CD45.1 (PE) | 110708 |
|  | Anti-mouse CD45.2 (BV421) | 109831 |
|  | Anti-mouse CD45.2 (FITC) | 109806 |
|  | Anti-mouse CD45.2 (APC) | 109813 |
|  | Anti-mouse H-2D^d^ (Alexa Fluor 647) | 110612 |
|  | Anti-human CD3 (PE/Cyanine7) | 317334 |
|  | Anti-human CD4 (BV421) | 357424 |
|  | Anti-human CD8 (FITC) | 300906 |
|  | Anti-human CD56 (PE) | 985902 |
|  | Anti-human Ki67 (APC) | 350514 |
|  | Rat IgG2a, κ isotype control (PE/Cyanine7) | 400522 |
|  | Rat IgG2a, κ isotype control (FITC) | 400506 |
|  | Mouse IgG1, κ isotype control (APC) | 400122 |
|  | Donkey anti-rabbit IgG (Alexa Fluor 647) | 406414 |
|  | Cyto-Fast^TM^ Fix/Perm buffer | 426803 |
|  | Fixation buffer | 420801 |
|  | Intracellular Staining Perm Wash Buffer | 421002 |
|  | True-Nuclear^TM^ transcription factor buffer set | 424401 |
|  | Annexin V Binding Buffer | 422201 |
|  | Mouse recombinant IL-33 | 580506 |
|  | Human recombinant IL-33 | 581804 |
|  | Purified anti-human CD3 antibody | 317302 |
| PeproTech | Mouse recombinant IL-2 | 200-02 |
| (NJ, USA) | Human recombinant IL-2 | 212-12 |
| Cell Signaling Technology | Bcl-xl (54H6) (PE) | 13835S |
| (MA, USA) | Bcl-xl (54H6) | 2764S |
|  | Survivin (71G4B7) (PE) | 5875S |
|  | Survivin (71G4B7) | 2808S |
|  | P-IKKα/β | 2697S |
| Abcam | Anti-NF-κB (P105/P50) | ab32360 |
| (Cambridge, MA, USA) | Anti-IκBα (phospho S36) | ab133462 |
|  | Goat anti-rabbit IgG (Alexa Fluor 488) | ab150077 |
| Beyotime (Shanghai, China) | GreenNuc^TM^ Caspase-3 Assay Kit | C1168M |
| Miltenyi Biotec | Mouse T cell activation/expansion kit | 130-093-627 |
| (Auburn, CA) | Human double negative T cell isolation kit | 130-092-614 |
| Thermo Fisher Scientific | BrdU staining kit | 8817-6600-42 |
| (MA, USA) | Cell Trace^TM^ Violet Cell Proliferation Kit | C34557 |

***Table S2 Primers used in this study***

|  |  |  | Sequence (5'→3') | |
| --- | --- | --- | --- | --- |
| Bcl-2 | Mouse | Sense | | TGAGTACCTGAACCGGCATCT |
|  |  | Anti-sense | | GCATCCCAGCCTCCGTTAT |
| Bcl-xl | Mouse | Sense | | AACATCCCAGCTTCACATAACCCC |
|  |  | Anti-sense | | GCGACCCCAGTTTACTCCATCC |
| Survivin | Mouse | Sense | | GCTTCATCCACTGCCCTAC |
|  |  | Anti-sense | | GTGCTTTCTATGCTCCTCTAT |
| Bax | Mouse | Sense | | GCGTGGTTGCCCTCTTCTACTTTG |
|  |  | Anti-sense | | AGTCCAGTGTCCAGCCCATGATG |
| Bik | Mouse | Sense | | ACGTGGACCTCATGGAGTG |
|  |  | Anti-sense | | TGTGTATAGCAATCCCAGGCA |
| St6gal1 | Mouse | Sense | | CTCCTGTTTGCCATCATCTGC |
|  |  | Anti-sense | | GGGTCTTGTTTGCTGTTTGAGA |
| Bcl2l11 | Mouse | Sense | | GAGATACGGATTGCACAGGA |
|  |  | Anti-sense | | ATTTGAGGGTGGTCTTCAGC |
| Nr4a3 | Mouse | Sense | | TGCGTGCAAGCCCAGTATAG |
|  |  | Anti-sense | | ATAAGTCTGCGTGGCGTAAGT |
| Casp7 | Mouse | Sense | | AAGACGGAGTTGACGCCAAG |
|  |  | Anti-sense | | CCGCAGAGGCATTTCTCTTC |
| Kit | Mouse | Sense | | GCCACGTCTCAGCCATCTG |
|  |  | Anti-sense | | GTCGCCAGCTTCAACTATTAACT |
| Ndrg1 | Mouse | Sense | | ATGTCCCGAGAGCTACATGAC |
|  |  | Anti-sense | | CCTGCTCCTGAACATCGAACT |
| Egr1 | Mouse | Sense | | TCGGCTCCTTTCCTCACTCA |
|  |  | Anti-sense | | CTCATAGGGTTGTTCGCTCGG |
| Sigirr | Mouse | Sense | | GTGACATGGCCCCTAATTTCC |
|  |  | Anti-sense | | ATGCCAGACCATCTTTCAGCC |
| Myd88 | Mouse | Sense | | TGGCCTTGTTAGACCGTGA |
|  |  | Anti-sense | | AAGTATTTCTGGCAGTCCTCCTC |
| Irak2 | Mouse | Sense | | GGAAGCCGGTTCCTGAGAG |
|  |  | Anti-sense | | GGCCGGACTTTCTCCTGTTC |
| Irak1 bp1 | Mouse | Sense | | AGCCGAGGTCTGCATTACATT |
|  |  | Anti-sense | | TGGCAGTCTGGATAACTGATGA |
| Traf4 | Mouse | Sense | | CCGGCTTCGACTACAAGTTC |
|  |  | Anti-sense | | TCAGGGCATTTGAAGACTCC |
| Traf6 | Mouse | Sense | | ATCCATAAGGGATGCAGGGC |
|  |  | Anti-sense | | GGCACTTTACCGTCAGGGAA |
| RelA | Mouse | Sense | | GGATGGCTACTATGAGGCTGAC |
|  |  | Anti-sense | | AGGTCTCGCTTCTTCACACACT |
| P50 | Mouse | Sense | | GTGACAGTGGTGTGGAGACATC |
|  |  | Anti-sense | | GGGGCATTTTGTTCAGAGATAG |
| Perforin | Mouse | Sense | | CTGCCACTCGGTCAGAATG |
|  |  | Anti-sense | | CGGAGGGTAGTCACATCCAT |
| Granzyme B | Mouse | Sense | | GGAACACCTCTTCTGCCACC |
|  |  | Anti-sense | | AGCATTAGATAACATTCTCGGGG |
| Fasl | Mouse | Sense | | TGAATTACCCATGTCCCCAG |
|  |  | Anti-sense | | AAACTGACCCTGGAGGAGCC |
| CD39 | Mouse | Sense | | AGATGAAATCGGTGCG |
|  |  | Anti-sense | | GGTATCAGTTCGGTGGA |
| Pd1 | Mouse | Sense | | CGTCCCTCAGTCAAGAGGAG |
|  |  | Anti-sense | | GTCCCTAGAAGTGCCCAACA |
| Klrb1a | Mouse | Sense | | TGGGAGGAAGGTCTAGTTGATTG |
|  |  | Anti-sense | | TCTCCTGAGATAGCAGCACAG |
| Cd160 | Mouse | Sense | | GGGGCTAATACTCTTCTGGTGC |
|  |  | Anti-sense | | CTTTTCAGTGATGCCATCTGTCT |
| GAPDH | Mouse | Sense | | AAGGTCATCCCAGAGCTGAA |
|  |  | Anti-sense | | CTGCTTCACCACCTTCTTGA |
| Bcl-2 | Human | Sense | | GGTGGGGTCATGTGTGTGG |
|  |  | Anti-sense | | CGGTTCAGGTACTCAGTCATCC |
| Bcl-xl | Human | Sense | | GAGCTGGTGGTTGACTTTCTC |
|  |  | Anti-sense | | TCCATCTCCGATTCAGTCCCT |
| Survivin | Human | Sense | | AGGACCACCGCATCTCTACAT |
|  |  | Anti-sense | | AAGTCTGGCTCGTTCTCAGTG |
| Bax | Human | Sense | | CCCGAGAGGTCTTTTTCCGAG |
|  |  | Anti-sense | | CCAGCCCATGATGGTTCTGAT |
| Bik | Human | Sense | | GACCTGGACCCTATGGAGGAC |
|  |  | Anti-sense | | CCTCAGTCTGGTCGTAGATGA |
| Irak2 | Human | Sense | | CCAGGCAACCGATGACTTCAA |
|  |  | Anti-sense | | TGGGGTGGCAGCATCTAAGA |
| Irak4 | Human | Sense | | CTTGGATGGTACTCCACCACT |
|  |  | Anti-sense | | AAAATTGATGCCATTAGCTGCAC |
| Myd88 | Human | Sense | | GGCTGCTCTCAACATGCGA |
|  |  | Anti-sense | | CTGTGTCCGCACGTTCAAGA |
| Traf6 | Human | Sense | | TTGCCATGAAAAGATGCAGAGG |
|  |  | Anti-sense | | AGCCTGGGCCAACATTCTC |
| RelA | Human | Sense | | CCCACGAGCTTGTAGGAAAGG |
|  |  | Anti-sense | | GGATTCCCAGGTTCTGGAAAC |
| P50 | Human | Sense | | AACAGAGAGGATTTCGTTTCCG |
|  |  | Anti-sense | | TTTGACCTGAGGGTAAGACTTCT |
| IKKα | Human | Sense | | ATGAAGAAGTTGAACCATGCCA |
|  |  | Anti-sense | | CCTCCAGAACAGTATTCCATTGC |
| IKKβ | Human | Sense | | CTGGCCTTTGAGTGCATCAC |
|  |  | Anti-sense | | CGCTAACAACAATGTCCACCT |
| IκBα | Human | Sense | | CTCCGAGACTTTCGAGGAAATAC |
|  |  | Anti-sense | | GCCATTGTAGTTGGTAGCCTTCA |
| Perforin | Human | Sense | | GGCTGGACGTGACTCCTAAG |
|  |  | Anti-sense | | CTGGGTGGAGGCGTTGAAG |
| Granzyme B | Human | Sense | | TACCATTGAGTTGTGCGTGGG |
|  |  | Anti-sense | | GCCATTGTTTCGTCCATAGGAGA |
| Fasl | Human | Sense | | ATTTAACAGGCAAGTCCAACTCA |
|  |  | Anti-sense | | GGCCACCCTTCTTATACTTCACT |
| CD39 | Human | Sense | | AGGTGCCTATGGCTGGATTAC |
|  |  | Anti-sense | | CCAAAGCTCCAAAGGTTTCCT |
| CD73 | Human | Sense | | CCAGTACCAGGGCACTATCTG |
|  |  | Anti-sense | | TGGCTCGATCAGTCCTTCCA |
| OX40 | Human | Sense | | GCAATAGCTCGGACGCAATCT |
|  |  | Anti-sense | | GAGGGTCCCTGTGAGGTTCT |
| Tim-3 | Human | Sense | | AGACAGTGGGATCTACTGCTG |
|  |  | Anti-sense | | CCTGGTGGTAAGCATCCTTGG |
| NKG2A | Human | Sense | | AGCTCCATTTTAGCAACTGAACA |
|  |  | Anti-sense | | CAACTATCGTTACCACAGAGGC |
| NKG2D | Human | Sense | | CCTTGACCGAAAGTTACTGTGG |
|  |  | Anti-sense | | GGCTGGCATTTTGAGACATACAA |
| GAPDH | Human | Sense | | GGAGCGAGATCCCTCCAAAAT |
|  |  | Anti-sense | | GGCTGTTGTCATACTTCTCATGG |
